# Calcium interactions in amelogenin-derived peptide assembly

**DOI:** 10.3389/fphys.2022.1063970

**Published:** 2022-12-14

**Authors:** Jing Zhang, Yushi Bai, Jian Wang, Bing Li, Stefan Habelitz, Jun-xia Lu

**Affiliations:** ^1^ School of Life Science and Technology, ShanghaiTech University, Shanghai, China; ^2^ University of Chinese Academy of Sciences, Beijing, China; ^3^ State Key Laboratory of Molecular Biology, CAS Center for Excellence in Molecular Cell Science, Shanghai Institute of Biochemistry and Cell Biology, Chinese Academy of Sciences, Shanghai, China; ^4^ Department of Preventative and Restorative Dental Sciences, School of Dentistry, University of California, San Francisco, San Francisco, CA, United States

**Keywords:** amelogenin, assembly, mineral ions, phosphorylation, SSNMR

## Abstract

Phosphorylation of serine residues has been recognized as a pivotal event in the evolution of mineralized tissues in many biological systems. During enamel development, the extracellular matrix protein amelogenin is most abundant and appears to be critical to the extreme high aspect ratios (length:width) of apatite mineral fibers reaching several millimeters in larger mammalian teeth. A 14-residue peptide (14P2, residues Gly8 to Thr21) was previously identified as a key sequence mediating amelogenin assembly formation, the domain also contains the native single phosphoserine residue (Ser16) of the full-length amelogenin. In this research, 14P2 and its phosphorylated form (p14P2) were investigated at pH 6.0 with various calcium and phosphate ion concentrations, indicating that both peptides could self-assemble into amyloid-like conformation but with differences in structural details. With calcium, the distance between ^31^P within the p14P2 self-assemblies is averaged to be 4.4 ± 0.2Å, determined by solid-state NMR ^31^P PITHIRDS-CT experiments. Combining with other experimental results, solid-state Nuclear Magnetic Resonance (SSNMR) suggests that the p14P2 self-assemblies are in parallel in-register *β*-sheet conformation and divalent calcium ions most likely connect two adjacent peptide chains by binding to the phosphate group of Ser16 and the carboxylate of Glu18 side-chain. This study on the interactions between calcium ions and amelogenin-derived peptides provides insights on how amelogenin may self-assemble in the presence of calcium ions in early enamel development.

## 1 Introduction

**FIGURE 1:**
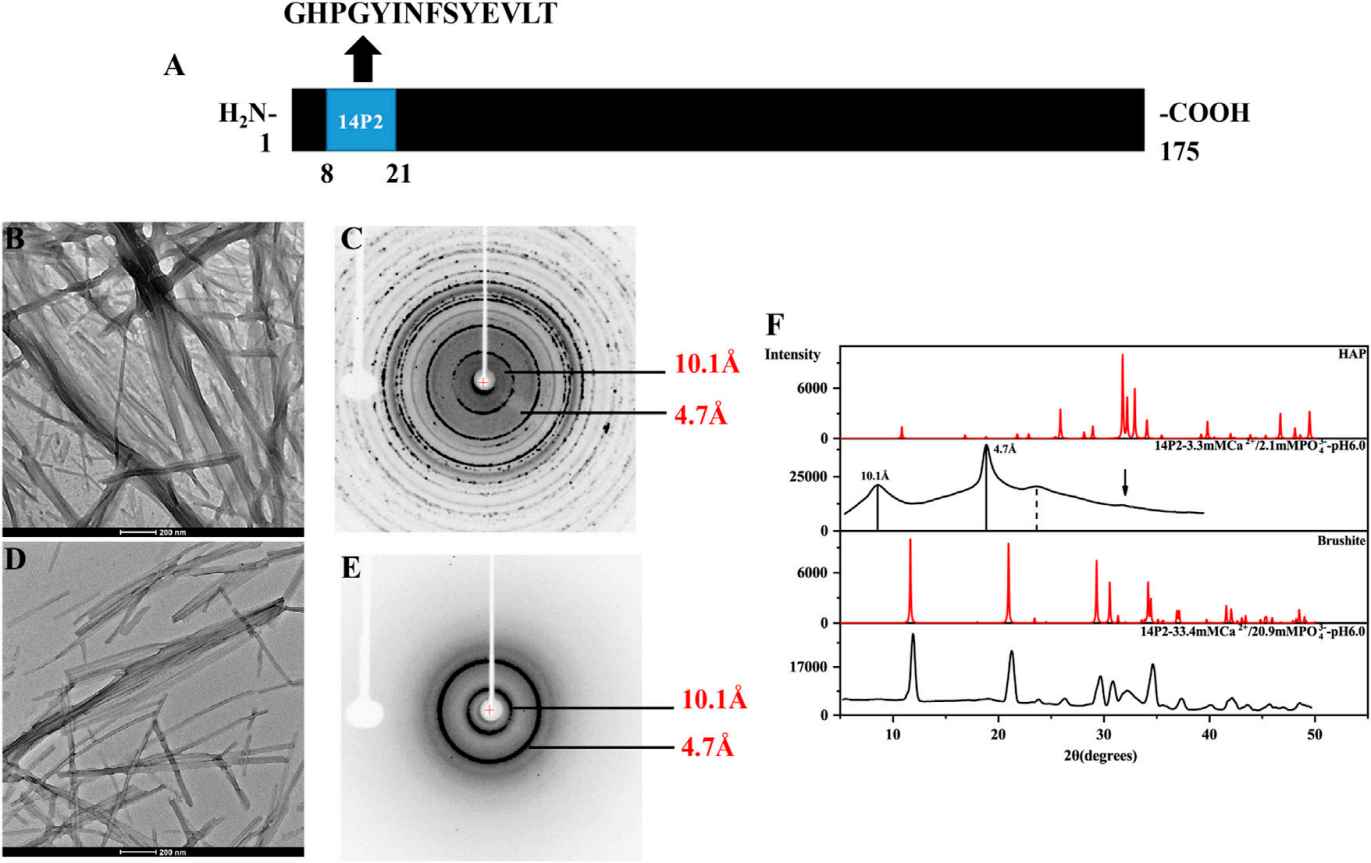
TEM images and the X-ray diffraction of the 14P2 self-assembly formed at pH 6.0 with different concentration of calcium and phosphate ions. **(A)** The location of 14P2 sequence in H175; **(B,C)** were obtained for the sample prepared in the presence of high concentration of calcium (33.4 mM) and phosphate ions (20.9 mM), **(D,E)** were obtained for the sample prepared in the presence of low concentration of calcium (3.3 mM) and phosphate ions (2.1 mM). For **(B)**, aliquots of sample suspension were taken for TEM after the peptide was incubated in the solution for 12 days; For **(D)**, the peptide was incubated for 9 days before aliquots of solution was taken for TEM; **(C,E)** were the X-ray diffraction images of 14P2 assembly, all aggregates were collected by centrifugation. The marked rings indicate the equatorial and meridional diffractions at 10.1 and 4.7 Å, respectively. **(C)** Also displayed many other Debye rings, indicating mineral salt formation; **(F)** comparison of the powder diffractogram of the peptide 14P2 assembly with the crystal hydroxyapatite (HAP) and brushite. The arrow indicated a small mineral diffraction peak for the sample prepared at low concentration of ions.

In natural proteins, phosphorylated serine often plays a role in binding mineral ions (Addison et al., 2013). For example, Dentin Phosphoprotein (DPP) and Dentin Matrix Protein-1 (DMP-1) contain a large number of aspartic acids and phosphoserines (Alvares, 2014) and the interaction with calcium serves as the first step to recruit the mineral ions for subsequent mineralization of collagen fibrils (Prasad et al., 2010). Another example is the silk spun by caddisfly underwater which contains phosphorylated serine-rich motifs that enable the silk to adhere to rocks (Addison et al., 2013). Amelogenin is the most abundant protein in the developing enamel matrix. It is believed that the protein matrix guides the mineral crystal deposits and results in the highly organized structure of apatite nanofibers which provide tooth enamel with extraordinary mechanical properties (Lacruz et al., 2017). However, how the protein matrix is organized in enamel mineral nucleation is still a debate. It has been shown that amelogenin could assemble into aggregates with *β*-amyloid properties in the presence of calcium and phosphate ions (Martinez Avila et al., 2012; Carneiro et al., 2016; Engelberth et al., 2018; Ma et al., 2019; Zhang et al., 2020). A short sequence at amelogenin N-terminal domain from residue Gly8 to Thr21 (GHPGYINFSYEVLT, 14P2, Figure 1A) has been recognized for having a strong propensity to self-associate into amyloids (Carneiro et al., 2016). The residue numbering is based on the 175-residue full-length human amelogenin (H175). NMR studies on protein aggregates formed by recombinant full-length human amelogenin also identified a region with *β*-strand secondary structure that overlap with 14P2 (Zhang et al., 2020). Interestingly, the same segment contains the single phosphorylated site (Ser16) in the native full-length human amelogenin (Takagi et al., 1984; Fincham et al., 1999). Different from the high calcium-binding ability provided by the dense population of phosphorylated serine in local regions in silk protein of caddisfly and in phosphoproteins of bone and dentin with dozens of phosphorylation site, the single phosphorylated residue in amelogenin is expected to play less of a role in directing enamel mineralization (Wiedemann Bidlack et al., 2011; Carneiro et al., 2016; Shin et al., 2020; Stifler et al., 2022), but may be critical in regulating protein structures and protein-protein interactions (Le Norcy et al., 2011; Wiedemann Bidlack et al., 2011; Carneiro et al., 2016). It was reported that the ratio of phosphorylated to all (phosphorylated + non-phosphorylated) amelogenin and its fragments was highest in superficial secretory and early maturation stage enamel and dephosphorylation of amelogenin was important for enamel maturation when the amorphous calcium phosphate gradually transits to crystalline apatite (Green et al., 2019).

Although amyloid is usually associated with cell toxicity and neurodegeneration, many functional amyloids have also been discovered these years playing key roles in regulating biosynthetic pathways (Fowler et al., 2007; Wu et al., 2021). The amyloid-like assemblies formed by the full-length amelogenin have proved to be able to template apatite crystal mineralization *in vitro* (Bai et al., 2020; Akkineni et al., 2022). In this work, the self-assembly of 14P2 and phosphorylated 14P2 at Ser16 (p14P2) were investigated in different solution conditions with variations in calcium and phosphate ion concentrations, since both ions are essential to enamel formation (Aoba and Moreno, 1987) and the concentrations of ions may have an effect on the status of protein assembly (Martinez Avila et al., 2012; Carneiro et al., 2016; Engelberth et al., 2018). Solid-state Nuclear Magnetic Resonance (SSNMR) is a powerful technique that is capable of studying proteins in non-crystalline solid or gel state, which is not possible with traditional protein structural characterization methods, such as x-ray crystallization and solution-NMR. NMR chemical shifts are sensitive to the nuclei’s coordination states and the local structure. SSNMR homonuclear dipolar recoupling technique (PITHIRDS-CT (Tycko, 2007)) can be used to obtain the distance information between the same type of nuclei, such as the distance between two neighboring ^13^C nuclei. SSNMR heteronuclear dipolar recoupling technique (REDOR (Gullion and Schaefer, 1989) can be used to obtain the distance information between two different types of nuclei, such as the distance between one ^13^C nucleus and one ^15^N nucleus. Here, SSNMR was utilized to study the assembled structures of 14P2 and p14P2 in combination with x-ray diffraction (XRD) and transmission electron microscopy (TEM) to gain a better understanding of calcium interations in amelogenin-derived peptide assembly.

## 2 Experimental section

### 2.1 Peptides self-assembly sample preparation

Peptide 14P2 (GHPGYINFSYEVLT) containing the sequence of the N-terminal domain (residues 8–21) of the human full-length amelogenin were synthesized. One site-specific isotopically labeled 14P2 peptide was synthesized, with labeling at Ile13 ^13^CO and Val19 ^15^NH. 14P2 peptide was purchased at ELIM Biopharmaceuticals, Inc. (CA, United States) and GL Biochem (Shanghai) Ltd. The peptide was dissolved in 0.01 M HCl first and diluted to a final concentration of 1 mg/ml using an aqueous solution containing different concentration of CaCl_2_ and KH_2_PO_4_. The experiments were performed on three conditions with the final calcium and phosphate ion concentration: 1) 33.4 mM calcium and 20.9 mM phosphate ions (high concentration condition); 2) 3.3 mM calcium and 2.1 mM phosphate ions (low concentration condition); 3) 33.4 mM calcium ions. Finally, the solution pH was slowly adjusted to 6.0 by adding 0.1 M KOH and the solution was incubated at 37°C for more than a week for peptide self-assembly for the first two samples. The last sample was incubated for only 2 days. Before the experiment, all the solution was purged using N_2_ gas about 2 h. During the experiment N_2_ gas was purged directly to the surface of the solution.

Meanwhile, the peptide with a phosphorylated Ser 16 (p14P2) was also synthesized and purchased from Elim Biopharmaceuticals, Inc. (CA, United States) and ChinaPeptides (Shanghai) Ltd. Five samples of peptide self-assembly were prepared with different solution conditions: 1) high concentration of calcium ions (33.4 mM) at pH 6.0; 2) low concentration of calcium ions (3.3 mM) at pH 6.0; 3) low concentration of calcium (3.3 mM) and phosphate ions (2.1 mM) at pH 6.0; 4) in absence of calcium and phosphate ions at pH 6.0; 5) 3.3 mM calcium ions at pH 7.5. The salt concentrations and the assembly conditions were according to the literature (Martinez Avila et al., 2012; Carneiro et al., 2016) and modified slightly.

### 2.2 X-ray diffraction (XRD)

All samples were harvested using centrifugation at 259,000 g for 1 h at room temperature (Optima Max-TL, BECKMAN COULTER, Bera, CA, United States). The precipitation contained the peptide self-assemblies aggregates in nanometer size with or without the mineral crystals. The sample was mounted on the XRD sample loop. The experiment was measured using a single crystal X-ray diffraction instrument (Bruker D8 VENTURE, Bruker, Karlsruhe, Germany) with Cu K*α* radiation at 0.154184 nm wavelength operating at an acceleration voltage of 50 kV and a current of 1 mA at 298 K. The exposure time was varied from 30 to 60 s with 50 mm distance from sample to the detector. The acquired images were processed with the adxv.x86_64RHEL6 program (Scripps Research Institute, La Jolla, CA, United States). And the data were further processed and integrated to be transformed into the powder pattern with X’Pert HighScore Plus software (PANalytical B.V., Almelo, Netherlands). The structural data of crystals hydroxyapatite (HAP) and brushite were taken from the ICSD database (The Inorganic Crystal Structure Database HAP-169498-ICSD, brushite-16132-ICSD).

### 2.3 Transmission electron microscopy (TEM)

The samples incubated for more than 7 days were taken out periodically and observed using TEM. 5 *μ*L drop of sample solution was pipetted onto a carbon-coated copper grid (300 meshes, Beijing Zhongjingkeyi Technology Co., Ltd., Beijing, China) for 1 min and dried with filter paper. Then the grid was quickly washed off with three drops of Milli-Q water. Subsequently, the grid was stained with 5 *μ*L of 2% uranyl acetate in water (w/v) for 10 s and the stain solution was blotted away quickly with filter paper. To get a better staining effect, another drop of 5 *μ*L 2% uranium acetate was loaded to the grid for 1 min and washed off with one drop of water again. Finally, the grid was dried in the air. The grids were imaged with Talos L120C Transmission Electron Microscope (FEI company, Brno, Czech Republic) at 120 kV.

### 2.4 Solid-state NMR experiments

All NMR experiments were carried out on a 16.4T (700 MHz, ^1^H frequency) Bruker AVANCE NEO spectrometer. ^13^C–^13^C and ^13^C-^15^N correlation spectra were collected with a 3.2 mm triple channel HCN Bruker probe, while a 3.2 mm HCP MAS probe was utilized to perform experiments involving ^31^P. Chemical shifts were externally referenced to DSS (40.48 ppm, using the ^13^C downfield peak of adamantane) for ^13^C. ^31^P chemical shifts were indirectly referenced relative to 85% H_3_PO_4_ (0 ppm), calculated with the ^31^P/^1^H chemical shift ratio of 0.404807420. All the experiments were collected with a 2 s recycle delay.

^1^H-^13^C cross-polarization (CP/MAS) experiments were performed on the peptide samples with natural abundance ^13^C under 15 kHz Magic Angle Spinning (MAS). The experiments were conducted at 273K or 288K. The CP contact time was 1.6 m with ^1^H fields of 86.56 kHz, and the ^1^H 90° pulse was 3.1 *μ*s. During the acquisition, SPINAL-64 (Fung et al., 2000) ^1^H decoupling was also applied with a ^1^H field strength of 80.65 kHz.

1D ^1^H-^31^P CP and 2D ^1^H-^31^P HETCOR (Shi et al., 2013) experiments were acquired for all the samples at 273K with MAS speed of 20 kHz except for the condition in the absence of either ions. For that sample, the spectra were obtained with 15 kHz. The CP contact time was 1.7 ms with ^1^H fields of 89.07 kHz, and the ^1^H 90° pulse was 3.1 *μ*s. Decoupling of ^1^H was applied at a field of 80.65 kHz during acquisition. For extracting the ^31^P chemical shift anisotropy (CSA) parameters of p14P2 samples, the 1D ^1^H-^31^P CP spectra were obtained at multiple MAS speeds of 0, 5, and 15 kHz. 1D ^31^P spectra were analyzed using the program DMFIT (Massiot et al., 2002) with the Herzfeld-Berger convention (Herzfeld and Berger, 1980). The principle elements of the chemical shift tensor are the isotropic value (*δ*_*iso*_), the asymmetry (dCS) and anisotropic (etaCS) parameters defined by *δ*_*iso*_ = 1/3 (*δ*_33_+*δ*_22_+*δ*_11_), dCS =(*δ*_33_–*δ*_*iso*_) and etaCS=(*δ*_22_–*δ*_11_)/(*δ*_33_–*δ*_*iso*_), respectively.

The intermolecular ^13^C-^15^N distance was measured with REDOR (Gullion and Schaefer, 1989) experiment at MAS speed of 10 kHz at 268K. The experiments were tested with the lengths of 90° pulse for ^1^H, ^13^C and ^15^N 3 *μ*s, 3.5*μ*s and 4.6*μ*s, respectively. During the recoupling, XY-8 phase cycling was applied. ^1^H decoupling field strength was 83 kHz with SPINAL-64 decoupling. The data points were acquired at the dephasing time of 1, 5, and 100 ms. The corresponding dephased (S) signal and the reference (S0) signal were then calculated to get the ratio S/S0 for obtaining the ^13^C-^15^N distance.

The intermolecular ^13^C-^13^C and ^31^P-^31^P distances were obtained using PITHIRDS-CT (Tycko, 2007) experiments with 20 kHz MAS at 273 K. The length of *π* pulse for dipole-dipole recoupling was one-third of the rotor period, 16.67 *μ*s. ^1^H decoupling fields was 80.65 kHz both during the recoupling period and the signal detection period. PITHIRDS recoupling was applied for a total of 19.2 ms in the ^13^C homonuclear dipolar experiments with ^13^C rf fields of 30 kHz. And PITHIRDS recoupling was applied for a total of 9.6 ms in the ^31^P homonuclear dipolar experiments with ^31^P rf fields of 30 kHz. The initial peak intensity was acquired at the effective homonuclear dipolar recoupling time of 0 ms. The peak intensity would decay as a function of the effective recoupling time. The acquired data were further processed to fit the numerical simulations using the software SIMPSON (Bak et al., 2011) to get the homonuclear distance.

## 3 Results

### 3.1 14P2 peptide formed an amyloid with varied conformations influenced by the solution conditions

**SCHEME 1:**
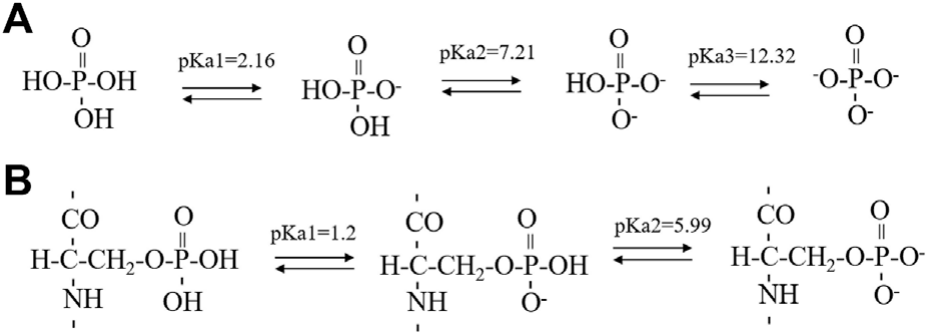
The successive steps of deprotonation of phosphoric acid **(A)** and phosphoserine **(B)**. The pKa1 value of phosphoserine was predicated by the Calculators Predictors from Chemaxon (https://chemaxon.com/products/calculators-and-predictorspka) while the pKa2 value of phosphoserine was obtained from literature using a five-residue peptide in a random conformation.

Synthetic 14P2 peptide, GHPGYINFSYEVLT, was first studied at two different solution conditions at pH 6.0. High-ion concentration condition contained 33.4 mM calcium and 20.9 mM phosphate ions, while the low-ion concentration was 3.3 mM calcium and 2.1 mM phosphate ions (Table 1). The high-ion concentration is used in order to determine a clear effect of ions on protein interactions and self-assembly structures, while the low-ion cencentraion is only slightly saturated towards brushite and close to the saturation level reported for the enamel fluid in the early secretory stage of porcine amelogenesis (Aoba and Moreno, 1987). 14P2 solution remained clear at low pH and turned cloudy at pH 6.0 (Supplementary Figure S1A). As previously shown, a pH of 6.0 or lower enhances ribbon formation and prevents spontaneous mineral precipitation. The Ka2 of phosphoric acid is 10^–7.21^. At pH 6.0, the more acidic form [H_2_(PO_4_)^−^] is prevelant but still solutions with high-ion concentration are saturated for brushite (CaHPO_4_⋅2H_2_O, Ksp = 10^–6.60^) (Scheme 1A, Supplementary Table S1 showed a Table of degree of saturation for two minerals). The cloudiness of both solution increased after several days of incubation at 37°C, but no mineral salt precipitations were visible by eye. More than 90% peptide would be in the pellets after centrifugation for both conditions, judged by the UV reading from the supernatant. The TEM images of both sample suspensions are displayed in Figures 1B, D. For the high salt condition, the image showed thick and big bundles of assemblies. For the low salt condition, it showed flat ribbon-like structures formed by the laterally associated thin filaments. No apparent twist could be observed in the assemblies for either sample. The XRD images of the total aggregates obtained by centrifugation were displayed in Figures 1C, E, showing two diffraction rings corresponding to 4.7 and 10.1 Å, the typical protein amyloid diffraction pattern. Therefore, 14P2 peptide assembled into amyloid structures at both the high-ion concentration and low-ion concentration conditions. However, the XRD image for the high-ion concentration condition was dominated by strong crystalline mineral diffractions, obscuring a clear observation of the protein amyloid diffraction. The mineral diffraction pattern of the high-ion concentration sample was consistent with the formation of brushite (CaHPO_4_⋅2H_2_O) (Figure 1F), consistent with the theoretical predication described above. However, no separate mineral particles could be observed by eye or TEM, suggesting the peptide binds tightly with the minerals forming organic-inorganic complexes. The XRD image for the low-salt condition was dominated by the peptide assembly diffraction, only showing very weak mineral diffraction peaks (Figure 1F, dark arrow), whereas the 1D ^1^H-^31^P cross polarization (CP) NMR spectrum of the sample pellet showed ^31^P mineral signals at 0.6 ppm and 2.9 ppm, indicating also the mineral formation (Supplementary Figure S2A). A 3.8 Å diffraction peak in Figures 1E, F was also observed, which was not identified. The presence of bisphosphonates was attributed to this diffraction peak in a previous publication (Sanii et al., 2014). Without phosphate ions, 14P2 can assemble at pH 6.0 in the presence of 33.4 mM calcium ions. The TEM images were obtained only after 2 days’ incubation, displaying both twisted and not twisted fibrils (Supplementary Figure S3).

**TABLE 1:** Protein self-assemblies, conditions and their structural properties.

Conditions	14P2	p14P2
33.4 mM [Ca^2+^] and 20.9 mM [P], pH = 6.0	Big bundles, parallel in-register amyloid	
33.4 mM [Ca^2+^], pH = 6.0	Twisted and flat fibrils, bundled, off-register amyloid	Twisted fibrils, bundled, in-register amyloid
3.3 mM [Ca^2+^] and 2.1 mM [P], pH = 6.0	Flat, shorter, ribbon-like, off-register amyloid	Twisted fibrils, bundled amyloid
3.3 mM [Ca^2+^], pH = 6.0		Twisted fibrils, bundled, in-register amyloid
3.3 mM [Ca^2+^], pH = 7.5		Twisted fibrils, bundled
0 mM [Ca^2+^] and 0 mM [P], pH = 6.0		Sphere

**FIGURE 2:**
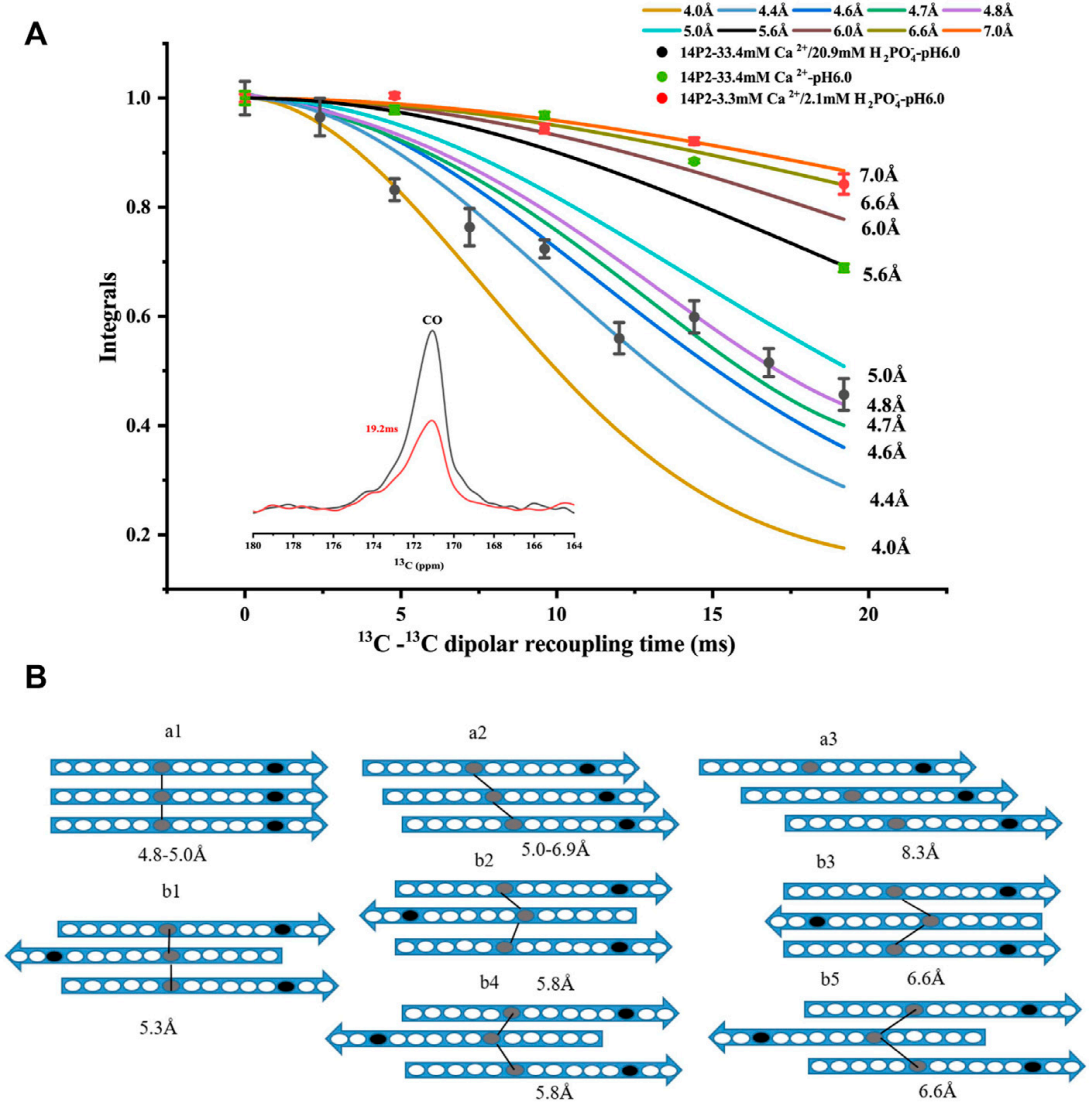
**(A)** ^13^C PITHIRDS-CT experiments measuring the intermolecular ^13^C-^13^C distances of the 14P2 assemblies. The 14P2 Ile13 ^13^CO and Val19 ^15^N were labeled for the samples with both calcium and phosphate ions. Only Ile13 ^13^CO was labeled for the sample with calcium ions but no phosphate. Comparison with the numerical simulations confirmed the 14P2 assemblies formed in the presence of 33.4 mM calcium and 20.9 mM phosphate adopted in-register parallel β-sheet structure with the intermolecular distance of 4.4 ± 0.4 Å, while the peptide assemblies formed in the low ion concentration displayed an intermolecular 13C-13C distance of 6.6 ± 0.4 Å. For 33.4 mM calcium without phosphate, the distance is 6.0 ± 0.6 Å. Each data point was taken by averaging of 2048 scans (1 h 9 min) for samples with both calcium and phosphate ions while 4000 scans (2 h 11 min) for 14P2 assembly formed with only calcium ions. **(B)** Structural models of the peptide assembly in parallel β-sheet conformation (a models) or antiparallel β-sheet conformation (b models), where gray oval represents Ile13 and black oval represents Val19. The lines were only shown for the distances within the SSNMR detection limits. The distances on the model were obtained from abeta fibrils in supplement Figure 4. Model a1 fit the distance of 4.4 ± 0.4 Å for the high-ion concentration condition (33.4 mM calcium and 20.9 mM phosphate) and model a2, b1–b5 fit the experimental constraints for 6.0 ± 0.6 Å or 6.6 ± 0.4 Å ambiguously.

In order to obtain more detailed structural information on the amyloid fibrils, the site-selective isotopically labeled peptides were synthesized for distance measurement using solid-state NMR. Since it was unknown whether the peptide adopted a parallel or antiparallel *β*-sheet conformation, two labels were utilized, one at Ile13 carbonyl carbon and the other at Val19 nitrogen, respectively (GHPGY(I^13^CO)NFSYE (V^15^N)LT). We hypothesized that if the peptide formed a parallel conformation, ^13^C-^13^C distance could be determined using solid-state NMR PITHIRDS-CT measurement (the detection limit is less than 8Å). On the other hand, if the peptide adopted an antiparallel conformation, the intermolecular ^13^C-^15^N distance may be within the range to be determined by solid-state NMR REDOR measurement (the detection limit is less than 7Å). Our results indicated that ^13^C-^15^N distance was too far to be determined for the conditions with both calcium and phosphate ions, however, ^13^C–^13^C distances were able to be determined for the two samples (Figure 2A). For condition 33.4 mM calcium ions without phosphate, only PITHIDRS-CT experiment was carried out. Our results showed the intermolecular I^13^CO distance was 4.4 ± 0.4Å for 14P2 peptide assemblies obtained at 33.4 mM calcium and 20.9 mM phosphate, 6.0 ± 0.6Å for assemblies prepared at 33.4 mM calcium without phosphate while the distance was 6.6 ± 0.4Å for the peptide assemblies obtained at 3.3 mM calcium and 2.1 mM phosphate. The distance was obtained by fitting the experimental data to the simulation values assuming ^13^C atoms were in a linear arrangement in the peptide assemblies with only a single distance. The 4.4 ± 0.4Å distance was close to the 4.7 Å obtained from XRD diffraction, consistent with an in-register parallel *β*-sheet conformation for the high salt concentration. Using the published A*β* fibril structure as a model (Supplementary Figure S4A), the parallel in-register structure (pdb:2LMN) displays a distance of 4.8–5.0 Å for two corresponding carbonyl carbon atoms at the adjacent peptides. For one-residue off-register parallel structure, there are two possibilities on the distance (5.07 Å and 6.84 Å between residue i and i+1 in this example). The schematic models were shown in Figure 2B, where model (a1) represented the in-register parallel *β*-sheet structure that fit the data best. However, the 6.0 ± 0.6Å or 6.6 ± 0.4Å distance was different from the XRD result. It indicated that although the peptide assemblies formed amyloid-like conformation, the backbone ^13^CO-^13^CO distance for Ile13 was different from the distance between two adjacent *β*-strands. It fit the (a2) model in Figure 2B assuming a parallel *β*-sheet structure. But it was also possible to construct from these data an antiparallel structure. An antiparallel A*β* fibril structure is shown in Supplementary Figure S4B (pdb:2LNQ), which is a structure aligned at residue i. It showed similar ^13^CO-^13^CO distances (5.3Å–6.6Å) between residue i and i-2, residue i and i-1 and residue i and i. But the difference in the distances was not significant for us to support a confident structural model. Therefore, the registry was ambigious for the antiparallel case. We listed all the possible antiparallel models (b1-b5) at Figures 2B. Our results indicated 14P2 assembled into a parallel in-register conformation only at high ion concentration (33.4 mM calcium and 20.9 mM phosphate).

### 3.2 The morphology of p14P2 self-assemblies was different from 14P2 self-assemblies

The effect of serine phosphorylation on the 14P2 peptide self-assemble was also investigated. Five conditions (Table 1) were tested including two conditions without inorganic phosphate ions, but with different calcium concentrations (33.4 mM or 3.3 mM); one condition with both calcium and inorganic phosphate ions (3.3 mM calcium and 2.1 mM phosphate ions); and one condition without either ion. All the conditions above were at pH 6.0. Another condition at pH 7.5, 3.3 mM calcium was also tested to check the pH effect on p14P2 assembly. Interestingly, at 33.4 mM calcium condition, the p14P2 peptide solution turned cloudy when calcium ions were added at the acidic condition (Supplementary Figure S1B). When the pH reached 6.0, the solution stayed turbid (Supplementary Figures S1C, D). Therefore, the calcium ions had interactions with the organic phosphoryl group in the peptide at acid pH condition and some peptide assemblies may already have been formed at the acid condition. For other conditions, the p14P2 solution turned cloudy in the presence of calcium ions only at pH 6.0. Judged by the pKa values reported for the phosphoryl serine in a five-residue peptide with a random structure in solution (Scheme 1B) (Bienkiewicz and Lumb, 1999), the organic phosphoryl group could be dominant by the monoanionic (-OPO_3_H^−^) at pH 2–3. At pH 6, around pKa2, the organic phosphoryl group could have monoanionic and dianionic (- ${{\text{OPO}}_{3}}^{2-}$ ) forms equally populated. Both negatively charged forms of the phosphoryl group could interact with positively charged calcium ions.

**FIGURE 3:**
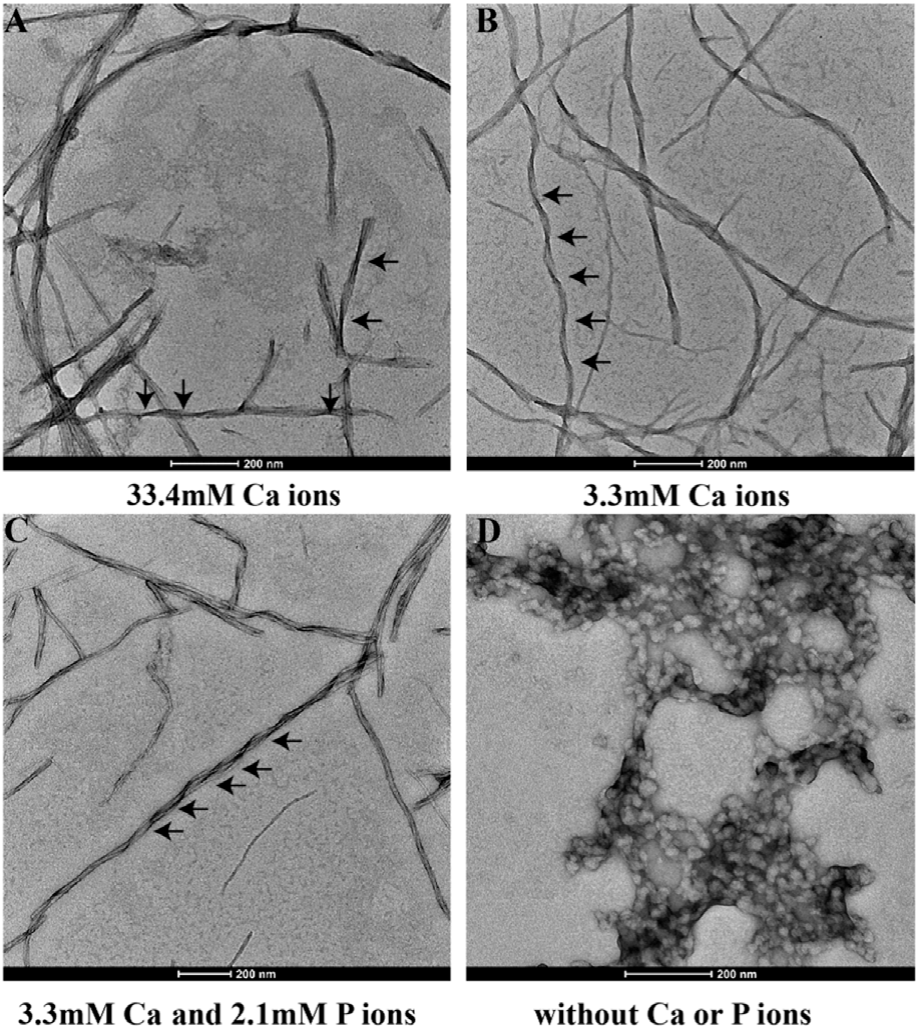
TEM images of p14P2 self-assemblies formed at pH 6.0 with or without calcium or phosphate ions. Aliquots of sample suspension were taken out after the sample was incubated for 14 days. The solution condition varied, **(A)** 33.4 mM calcium ions; **(B)** 3.3 mM calcium ions; **(C)** 3.3 mM calcium and 2.1 mM phosphate ions. **(D)** No calcium or phosphate ions.

**FIGURE 4:**
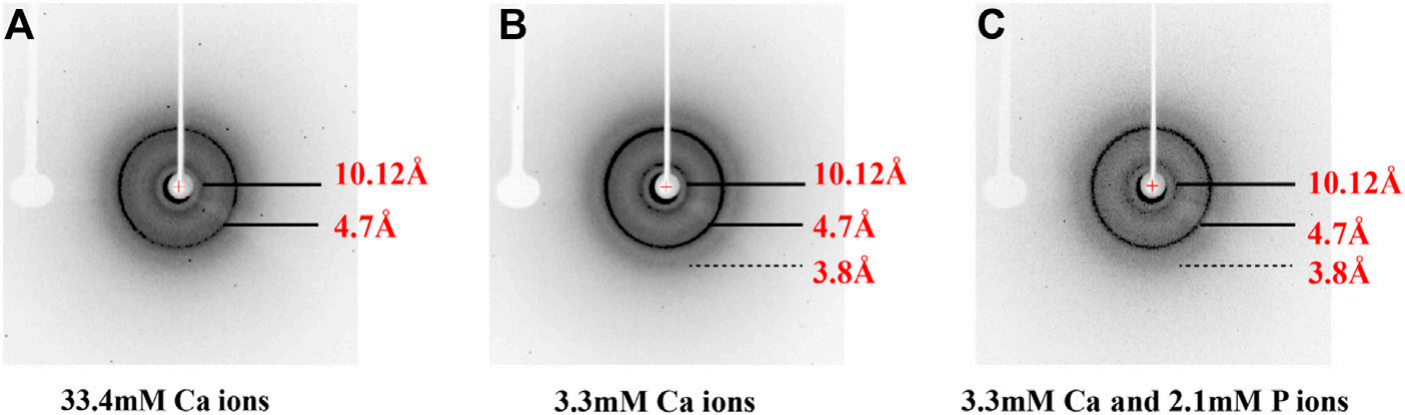
X-ray diffraction of peptide assemblies formed by incubating of p14P2 at pH 6.0 with various solution conditions. All the diffraction images illustrated the formation of the cross-*β* structure of p14P2 with the equatorial and meridional diffractions at 10.1 and 4.7 Å. The solution condition varied, **(A)** 33.4 mM calcium ions; **(B)** 3.3 mM calcium ions; **(C)** 3.3 mM calcium and 2.1 mM phosphate ions.

Figure 3 shows the TEM images of p14P2 self-assemblies. The samples were obtained from the solution suspension prepared using these four conditionsat pH6.0. The TEM image of the sample prepared at pH 7.5 was displayed at Supplementary Figure S5A. Different from 14P2, p14P2 assemblies in the presence of mineral ions at pH 6.0 all displayed twists with varied crossover lengths and width. The twisted fibril also tended to form super twists. When neither of the ions presented, the peptide formed less aggregates with mostly sphere structures (30–50 nm in diameter) (Figure 3D). At pH 7.5 condition, the assemblies showed less contrast in the TEM images. The XRD were also obtained for the p14P2 assemblies. The samples were collected using high-speed centrifugation, including all the aggregates formed in the preparation. All samples obtained at pH 6.0 displayed two dominant diffraction rings at 4.7 and 10.1 Å (Figure 4), supporting *β*-amyloid formation of the peptide. The sample prepared at pH 7.5 displayed the diffraction rings at 4.7 and 11.4 Å among all the distances shown in Supplementary Figure S5B. The result indicated that the *β*-amyloid conformation was maintained at pH 7.5 although the structures were not exactly the same for the two pH conditions. Additionally, in the presence of both calcium ions and inorganic phosphates (Figure 4C), no mineral diffraction was observed while p14P2 assemblies were formed.

### 3.3 The different organic phosphate states in the p14P2 amyloid by ^31^P-NMR analysis

**FIGURE 5:**
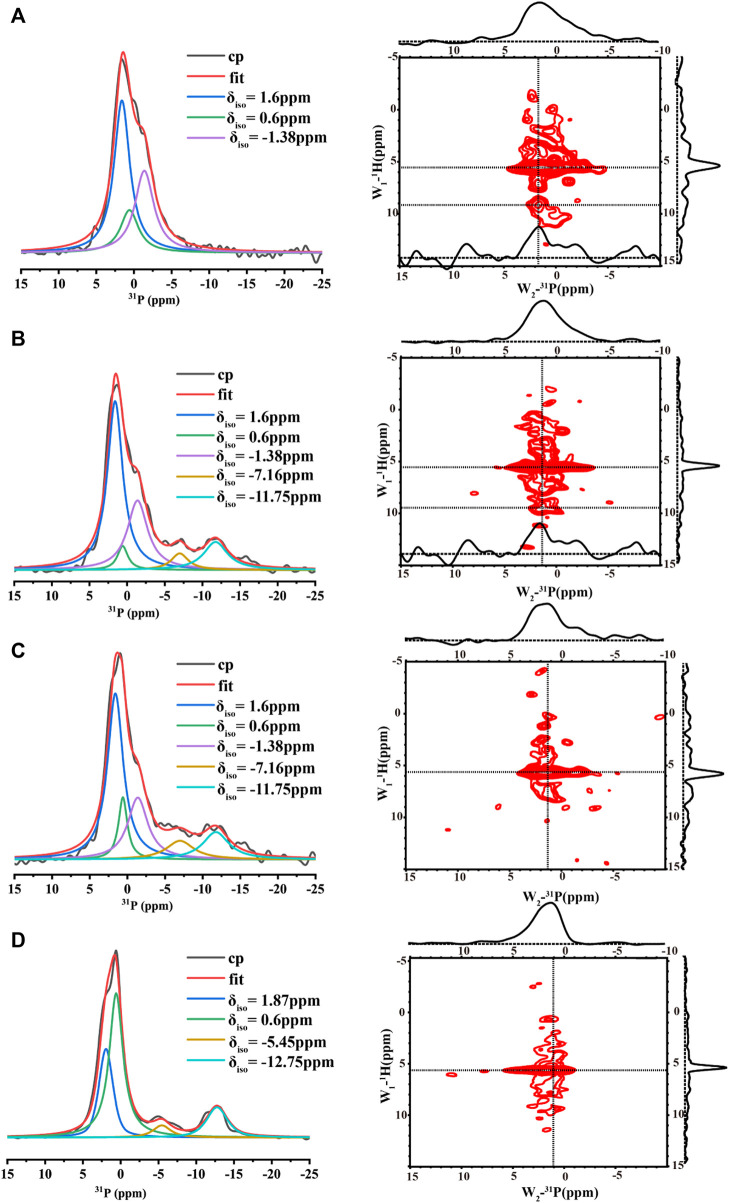
1D ^1^H- ^31^P CP MAS NMR spectra (left, each spectrum took about 2 h) and 2D ^1^H- ^31^P HETCOR spectra (right, each spectrum took about 6 h) acquired for p14P2 peptide assemblies formed at pH6.0 with different solution conditions. All the 1D spectra were deconvoluted to several components using DMFIT program with Gaus/Lor model. The raw data were shown in black, and the sum of all components from fitting were shown in red. Each component was labeled by its resonance position *δ*_*iso*_. The solution condition was varied, **(A)** 33.4 mM calcium ions; **(B)** 3.3 mM calcium ions; **(C)** 3.3 mM calcium and 2.1 mM phosphate ions. **(D)** No calcium or phosphate ions. All the spectra were taken at 20 kHz MAS except **(D)**. The spectra in **(D)** were taken at 15 kHz MAS.

The 1D ^1^H-^31^P CP spectra of the samples at the four conditions at pH 6.0 were also compared (Figure 5). All the spectra displayed broad lines composed of many peaks, suggesting multiple organic phosphate states. In order to understand each phosphate species, the spectra were deconvoluted using DMFIT(Massiot et al., 2002). Figure 5D shows the spectrum from the control sample without any calcium and inorganic phosphate ions. One peak around -12 ppm was close to the chemical shift of a pyrophosphate group (Yu et al., 2019), suggesting the peptides assembled into an in-register conformation that two neighboring phosphate groups on the serine were close enough to form the phosphoanhydride bond. However, the peak around -12 ppm represented only one of the components. The spectrum displayed one major peak at 0.6 ppm and a shoulder at 1.87 ppm. The major peak should represent free organic phosphate without binding to mineral ions and without interacting with other organic phosphate. Consistently, adding calcium ions (3.3 mM) decreased the peak intensities at 0.6 ppm, two additional peaks at 1.6 ppm and −1.38 ppm appeared (Figures 5B, C). At the high calcium ion concentration of 33.4 mM, the peak around −12 ppm disappeared while peaks at 1.6 ppm, −1.38 ppm dominated (Figure 5A). For all the spectra with calcium ions (Figures 5A–C), the peak at 0.6 ppm remained, but with much lower intensity compared to Figure 5D without calcium and inorganic phosphate ions. Together, these observations suggest that the peaks at 1.6 ppm and −1.38 ppm represent two major species of calcium-organic phosphate complexes. The 2D ^1^H-^31^P hetero-nuclear correlation (HETCOR) were displayed parallel to the 1D^31^P spectra. The 1D slices of the 2D spectra are displayed above each 2D spectrum and on the right side of the 2D spectrum. The 2D spectra for samples with calcium ions (right side of Figures 5A–C) all showed the water ^1^H correlation to the broad ^31^P peak at (5.5 ppm/∼1.6 ppm), indicating water molecules having a close interaction with the organic phosphate groups. For p14P2 assemblies without calcium ions, the correlation between water and the organic phosphate group at the serine residue was also observed as a broad peak at (5.5 ppm/∼1.0 ppm). At the same time, we can observe another weak correlation peak at around ^1^H/^31^P (9.5 ppm/1.6 ppm) for both 33.4 mM calcium and 3.3 mM calcium conditions. The low field (higher frequency) ^1^H peak was consistent with a protonated phosphate group (Leroy et al., 2017). The 1D slice at ^1^H 9.5 ppm position exhibited a narrower peak centered at ^31^P 1.6 ppm compared to the 1D slice at ^1^H 5.5 ppm showing above each 2D spectrum, suggesting that only the peak at ^31^P 1.6 ppm has a proton associated with it, but not the peak at −1.38 ppm.

**FIGURE 6:**
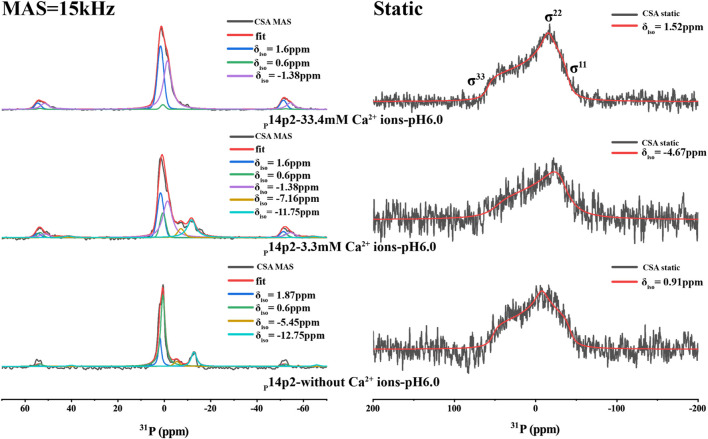
^1^H-^31^P CP spectra of p14P2 self-assembly formed at different solution conditions. The MAS spectra took about 6 h while the static spectra took about 15 h. The CSA parameters were obtained by fitting the spectra using DMFIT. And the fitting parameters are shown in Supplementary Table S2. The raw data are shown in black. The full DMFIT fitting spectra are shown in red. The spectra showed different resolved phosphorus resonances (*δ*_*iso*_) that can be ascribed to the different calcium binding status or different conformation of the single phosphorus site.

The chemical shift anisotropy (CSA) of ^31^P nuclei is very sensitive to its environment. It has been used as a probe to predict the organic phosphate ionization state and how they bind to calcium ions (Wu et al., 2003; Gardiennet Doucet et al., 2006; Addison et al., 2013). The CSA pattern of L-O-phosphoserine was reported in the literature (Addison et al., 2013), indicating a change of skew value [3 (*δ*_22_–*δ*_*iso*_)/(*δ*_11_–*δ*_33_)] of phosphoserine from positive to negative upon phosphoserine interactions with calcium or sodium cations. This is caused by the change of asymmetric CSA introduced to the phosphoserine environment in the interaction with mineral ions. In order to obtain the CSA parameters for p14P2 assemblies, 1D ^1^H-^31^P CP spectra at multiple magic-angle-spinning (MAS) speeds (15, 5 kHz and the static condition) were obtained and deconvoluted using DMFIT for 33.4 mM calcium and 3.3 mM calcium conditions (Figure 6 and Supplementary Figure S6). The results were summarized in Supplementary Table S2. The deconvolution on the 1D ^1^H-^31^P spectra of p14P2 amyloid at both calcium concentrations indicated that ^31^P peaks at 1.6 ppm and −1.38 ppm showed negative skew values, supporting that these two peaks were from calcium-organic phosphate complex. The peak at −1.38 ppm had a bigger CSA span compared to the peak at 1.6 ppm, indicating a less symmetric ^31^P nucleus environment. The peak at 0.6 ppm showed positive skew value, indicating this peak represents the organic phosphate component not binding to calcium ions. For the p14P2 self-assemblies formed without calcium and inorganic phosphate, the major peak at 0.6 ppm showed positive skew value, consistent with other samples. The information obtained from spectra at different MAS speeds generally agreed with each other at the sign of the skew values, but differed in the exact values because of the different experimental conditions. Our interpretation of CSA data is consistent with our conclusions obtained from Figure 5: the peak at 0.6 ppm represents the component not binding to calcium while the peak at 1.6 ppm represents single protonated phosphate group binding to calcium, the peak at −1.38 ppm represents dianionic phosphate group binding to calcium. A negative skew value was observed for the pyrophosphate peak at around −12 ppm, suggesting the chemical environment of the pyrophosphate bonding would also increase the asymmetry of ^31^P CSA. Other minor peaks were not discussed here. Interestingly, the organic phosphate groups was not saturated in binding to the calcium ions at 3.3 or 33.3 mM calcium ion concentration when the concentration of calcium ion was 5x or 50x of protein concentration. The existence of multiple organic phosphate peaks with different calcium coordination indicated the multiple modes of calcium binding to the phosphate group in p14P2.

The p14P2 sample prepared in the presence of both 3.3 mM calcium ions and 2.1 mM phosphate ions were different from others in that the sample contained both inorganic and organic phosphate groups (Table 1). The mineral ion concentrations in solution were the same for 14P2 self-assembly preparation at low ion concentrations. However, the morphology of the peptide self-assemblies was different from 14P2 assemblies (Figure 1C). The 1D ^1^H-^31^P CP spectra were also compared for the two types of peptide assemblies (Supplementary Figures S2A, B) with the same solution condition. The two 1D ^31^P spectra were very different, while the 1D ^1^H-^31^P CP spectra of p14P2 self-assemblies formed in 3.3 mM calcium with or without 2.1 mM phosphate ions were almost identical (Supplementary Figures S2B, C). Therefore, p14P2 assemblies prepared in the presence of both calcium and inorganic phosphate (Supplementary Figure S2B), appear to be free of calcium phosphate minerals. This indicates that the organic phosphate competed with the inorganic phosphate in the interaction with calcium ions for p14P2, reducing the calcium concentration in solution. Therefore, the organic phosphate group had a function of inhibiting calcium phosphate precipitation by reducing the free calcium concentration in solution. This observation is consistent with the full-length amelogenin or LRAP (Le et al., 2006), which binds about 4 to 6 calcium ions per protein molecule.

### 3.4 The amyloid formed by p14P2 adopted in-register conformations

**FIGURE 7:**
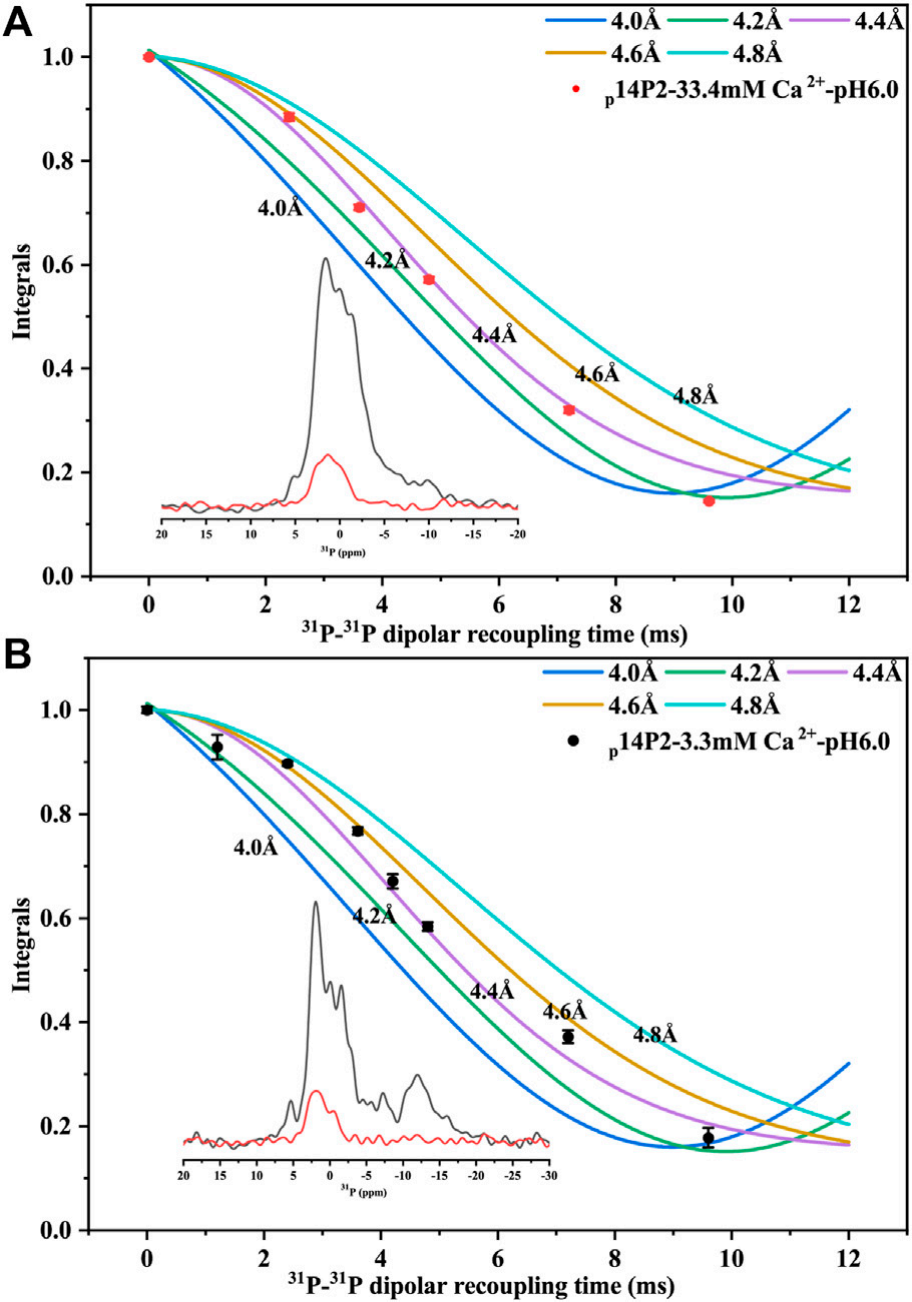
^31^P PITHIRDS-CT experiment for intermolecular ^31^P-^31^P distance measurement of p14P2 self-assemblies. The intermolecular ^31^P–^31^P magnetic dipole–dipole couplings were measured for p14P2 self-assemblies at pSer16. Comparison with numerical simulations confirmed the intermolecular distances of 4.4 ± 0.2 Å for both p14P2 assemblies formed in the high calcium concentration **(A)** and the low calcium concentration **(B)**, supporting an in-register parallel *β*-sheet structure of the assemblies. The peak area was integrated from −7.6 to 5.1 ppm for 33.4 mM Ca^2+^ condition **(A)** and from −4.2 to 4.7 ppm for 3.3 mM Ca^2+^ condition **(B)**. Each data point was from a NMR spectrum collected for 5 h and 35 min.

Solid-state NMR PITHIRDS-CT experiments were applied to study the conformation of the amyloid formed by p14P2. Since the peptide contained an organic ^31^P group, no other isotopic labeling was added. The intermolecular ^31^P-^31^P distance was measured using PITHIRDS-CT, shown in Figure 7 for samples with 33.4 or 3.3 mM calcium. The total peak area containing multiple components centered at 1.6 ppm was used, since it was difficult to deconvolute the peaks when the peak intensity became too small with the longer ^31^P-^31^P recoupling time. The PITHIRDS-CT data indicated a 4.4 ± 0.2 Å distance between two neighboring phosphate groups on the serine residues, generally consistent with an in-registry conformation of the peptide assembly. The distance was measured on the side-chain of the residues, which might explain the small deviation from the 4.7 Å distance for the peptide backbone determined using XRD. The twisted morphology of the fibril may make the side-chain phosphates closer. However, it is also possible that multiple ^31^P-^31^P distances exist, since the peak was broad with multiple components corresponding to different phosphoserine coordination states (corresponding to peaks at 1.6, −1.38, and 0.6 ppm).

**FIGURE 8:**
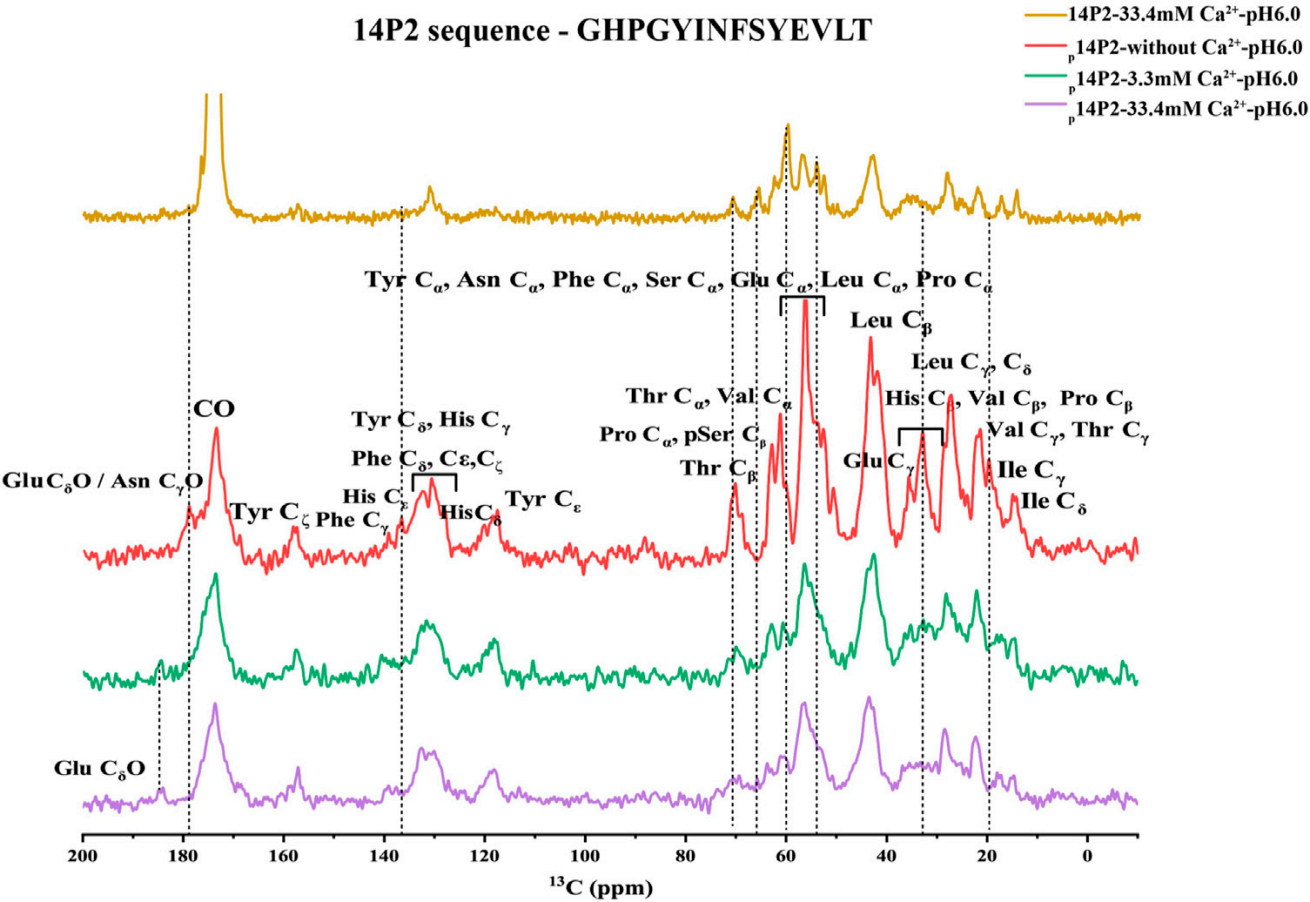
^1^H-^13^C CP-MAS NMR spectra of p14P2 and 14P2 assemblies. Spectra of p14P2 self-assemblies formed in 33.4 mM Ca^2+^ at pH 6.0 (purple, experimental time: 1 h 9 min), 3.3 mM Ca^2+^ at pH 6.0 (green, experimental time: 2 h 16 min), and no additional calcium or phosphate ions at pH 6.0 (red, experimental time: 1 h 9 min) and 14P2 assemblies formed at 33.4 mM calcium (gold, experimental time 5 h 40 min). The tentative assignment based on empirical values were labeled. The vertical lines (dashed line) were used to highlight some big differences for different samples.

The ^1^H-^13^C CP spectra of p14P2 assembly at 33.4 and 3.3 mM calcium concentration or without calcium were obtained in Figure 8 indicating smaller differences when comparing samples in the presence of calcium ions and bigger differences when comparing samples with or without calcium ions. The results confirmed that the major structure of the p14P2 assemblies was the same for the two conditions with different calcium ion concentrations. The spectra showed strong signals at the aromatic region; the C_*α*_, C_*β*_ regions of the phosphoserine and the methyl group positions for valine and isoleucine, consistent with a rigid structure of the assemblies. Interestingly, the side-chain carboxyl group signal for glutamic acid (Glu18) was also clearly observed around 184 ppm, indicating an ordered carboxyl group in the deprotonated state (Tollinger et al., 2002), suggesting this negatively charged side-chain may be involved in stabilizing the peptide assembly and calcium binding. Without calcium ions, the peak disappeared or shifted to a lower value (less than 180 ppm), supporting the influence of calcium binding on the chemical shift position of Glu18 side-chain carboxyl group. Furthermore, without calcium ions, the ^1^H-^13^C CP spectrum of p14P2 assembly also showed noticeable differences in other regions, such as Ile methyl group, Thr C_*β*_, Glu C_*γ*_ and His C_*β*_ regions. Together with the results described above, this observation indicated the central role of calcium ions in determining p14P2 assembly structures. The ^1^H-^13^C CP spectrum of 14P2 assemblies formed at 33.4 mM calcium concentration without any phosphate was also obtained to compare to p14P2 assembly spectra, which showed a bigger difference. For example, the highest resonance at Cɑ region was at around 60 ppm for 14P2 assemblies, where the p14P2 assemblies only showed weak peaks. The results supported our conclusion that phosphate group significantly influenced the peptide binding with the calcium ions and affected the peptide assembly structures.

## 4 Discussion

In this study, we observed the direct calcium interactions with the serine phosphorous group, manifested by a gradual change of ^31^P peaks of pSer16 in the NMR spectra upon adding calcium ions in the protein solution for the assembly (Figure 5). Furthermore, our ^13^C NMR results suggested the nearby Glu18 was involved in calcium binding. In the absence of calcium, the peptide agglomerates showed very different spectra, especially at the Glu side-chain carboxylate region, which has a very unique position and can be identified easily. This is consistent with a previous model of p14P2 proposed by Carneiro et al., which displayed intermolecular Ca^2+^ ions bridging from glutamate to phosphoserine (Carneiro et al., 2016).

The calcium content added was as high as 33.4 mM, 50x of the peptide or the organic phosphate concentration for p14P2, and thus much higher than observed under natural conditions. The purpose is to clearly demonstrate how the calcium ions affect the protein interactions and the structure of the protein assemblies. The twisted morphology was mostly observed for p14P2 peptide assemblies, however, a 14P2 assembly sample prepared without phosphate at 33.4 mM calcium displayed some twisted fibrils. Without phosphate, the high concentration of calcium ions can only interact with the peptide. The result suggested the calcium interaction with the peptide would promote the development of a twisted morphology for the fibrils.

For PITHIRDS-CT experiments, the experimental results were fit to the simulation curves, assuming a homogeneous structure model with a single distance. However, our sample may not be so homogeneous considering the linewidth for the carbonyl group was about 2 ppm. A distance of 4.4 ± 0.4 Å was obtained for a 14P2 peptide assembly at high mineral ion concentration, it suggested an in-register parallel conformation for 14P2. The distance is shorter than 4.7 Å expected for a parallel *β*-sheet structure, such as A*β* fibrils. The average intermolecular ^31^P-^31^P distance 4.4 Å also suggested an in-register parallel conformation for p14P2. It would need more distance information from other residues to get a confident answer. The twisted morphology of the fibril could be used to explain the short distance. The curve on the *β*-sheet surface might cause those phosphate groups to be closer. Also there may be multiple ^31^P-^31^P distances. For SSNMR dipolar-interation based distance measurement, the experimental results would mainly be determined by the shortest distances if multiple distances exist. Therefore, even if there are multiple distances, the distances that matter in the measurement should be very close to 4.4 Å. On the other hand, possibly, multiple ^31^P-^31^P distances within 5 Å co-exist but are located at separated structural units. In each unit, the phosphorylated serine side chain structure is unique and only a single ^31^P-^31^P distance exists. For example, one structure unit of the peptide assembly has a^31^P-^31^P distance of 4.1 Å and the other structure unit has a^31^P-^31^P distance of 4.7 Å. A combination of both could give a good fit for p14P2 assemblies prepared at 33.4 and 3.3 mM Ca^2+^ (Supplementary Figure S7). In this simulation, the contribution ratio of each structure unit was chosen to be close to the peak area ratio revealed by the deconvolution shown in Figure 5 (two peaks at 1.6 and −1.38 ppm). For p14P2 assemblies prepared at 3.3 mM Ca^2+^, a combination of 3.8 and 4.6 Å were also tested since 3.8 Å was a distance observed by XRD.

The peptide assemblies were obtained mainly at pH 6.0 condition, and the assemblies were harvested after 1 week incubation in this research. However, we also tested p14P2 at pH 7.5. And amyloid-like strucures were observed at 2 days. Therefore, the slightly acidic pH and the long incubation time were not necessary for the amyloid assembly formation. NMR is a low sensitive technique, requiring milligrams of isotopically labeled protein samples. A slightly acidic pH and a long incubation time were chosen in this research to promote the kinetics and give a higher yield of assemblies for NMR studies. On the other hand, slightly acidic pH may be of biological significance as pH values below 6 have been observed in the vicinity of the Tomes processes of ameloblasts and exocytosing vesicles are known to have pH around 5.5 (Sasaki et al., 1991; Lacruz et al., 2010).

We surveyed the ^31^P-^31^P distance in brushite and HAP crystal (Supplementary Figure S8), showing that the shortest ^31^P-^31^P distance for brushite is 3.8 Å while ^31^P-^31^P distances along the c axis for HAP are between 4.0 and 4.2 Å, very close to the experimental results, although here the experimental results were on the organic phosphate. These distances indicate that the organic phosphate-calcium interaction network would be a good starting point for the inorganic ions to interact with and to promote a phase transformation from amorphous calcium phosphate to crystalline mineral oriented with the organic supramolecular template (Akkineni et al., 2022). The organic phosphate interaction network could coordinate with the calcium ions, reduce the calcium ion concentrations in solution, and bring water molecules into proper positions, all of which are necessary for a controlled calcium mineralization process. Without calcium, the peptide assembly structure was very different. Table 1 lists all the peptide samples and their different structural properties. These results emphasized the importance of calcium and phosphoserine interactions in determining the modes of protein-protein interactions. Understanding these interactions may pave the way for us to understand the driving force for the self-assembly of full-length amelogenin and its ability to guide biomineralization in enamel.

## Data Availability

The original contributions presented in the study are included in the article/Supplementary Material, further inquiries can be directed to the corresponding authors.
