# Corrigendum: Calcium interactions in amelogenin-derived peptide assembly

**DOI:** 10.3389/fphys.2023.1178589

**Published:** 2023-04-04

**Authors:** Jing Zhang, Yushi Bai, Jian Wang, Bing Li, Stefan Habelitz, Jun-Xia Lu

**Affiliations:** ^1^ School of Life Science and Technology, ShanghaiTech University, Shanghai, China; ^2^ University of Chinese Academy of Sciences, Beijing, China; ^3^ State Key Laboratory of Molecular Biology, CAS Center for Excellence in Molecular Cell Science, Shanghai Institute of Biochemistry and Cell Biology, Chinese Academy of Sciences, Shanghai, China; ^4^ Department of Preventative and Restorative Dental Sciences, School of Dentistry, University of California, San Francisco, San Francisco, CA, United States

**Keywords:** amelogenin, assembly, mineral ions, phosphorylation, SSNMR

In the published article, there was an error in the Funding statement. We did not include funding received from an RO1 grant from the NIH/NIDCR.

The corrected Funding statement appears below.

“The work is supported by grants from the Natural Science Foundation of China (Nos 31770790 and 32171185 to J-XL) and the NIH/NIDCR (RO1DE025709, RO1DE031946 to SH).”

The authors apologize for this error and state that this does not change the scientific conclusions of the article in any way. The original article has been updated.

