# Supplementary material for: Calcium interactions in amelogenin-derived peptide assembly
Source: Front Physiol. 2022 Dec 14;13:1063970. doi: 10.3389/fphys.2022.1063970 (PMC9795176; doi:10.3389/fphys.2022.1063970)
Supplement: Supplementary file 1 [file DataSheet1.pdf]

Supplementary Material

1 Supplementary Figures and Tables

1.1 Supplementary Figures

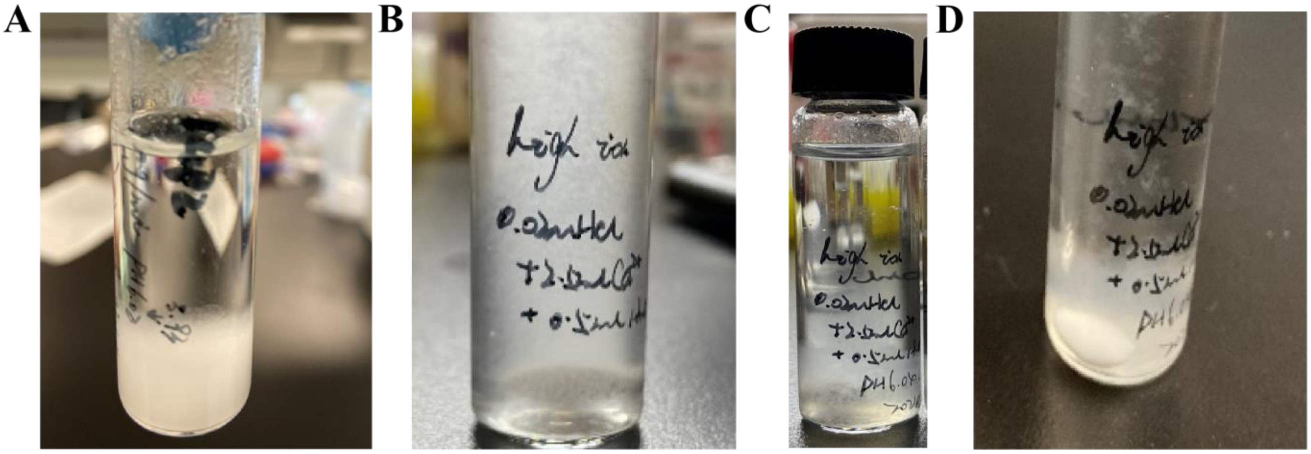

Supplementary Figure 1. The images of 14P2 and p14P2 peptide assembly samples. (A) was the 14P2 sample prepared in the presence of high concentration of calcium (33.4mM) and phosphate ions (20.9mM) and was incubated for a week; (B), (C) and (D) were the p14P2 samples prepared in the presence of high concentration of calcium (33.4mM) before the pH adjustment (B) and after the pH adjustment to 6.0 incubated for a day (C) and for a week(D).

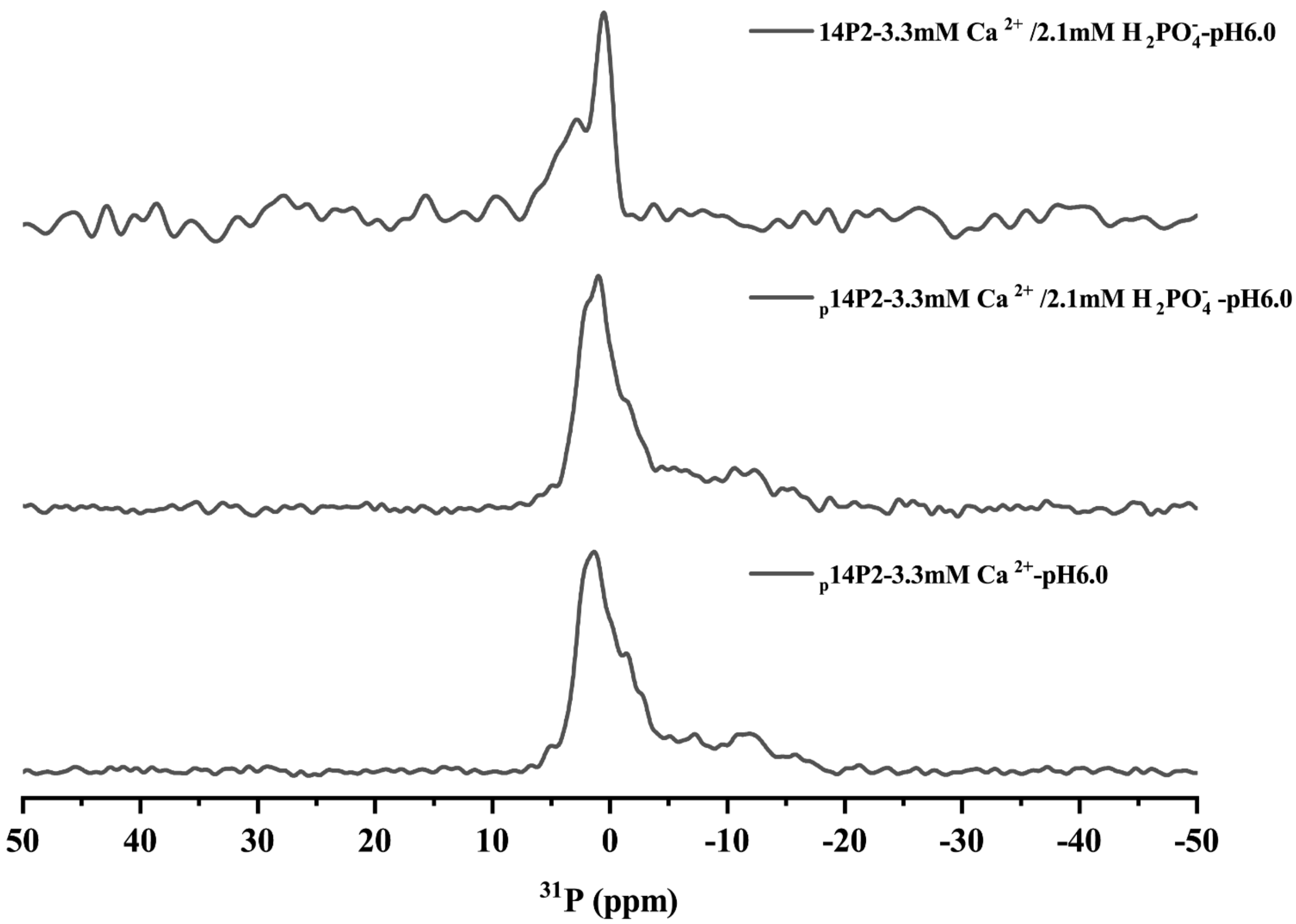

Supplementary Figure 2. ^{1}H-^{31}P CP-MAS NMR spectra of the 14P2 (A) and p14P2 (B) assembly formed in the presence of 3.3 mM calcium and 2.1 mM phosphate ions at pH 6.0. ^{1}H-^{31}P CP-MAS NMR spectrum of the p14P2 assembly formed in the presence of 3.3mM calcium but without phosphate was also shown for comparison (C). B, C spectra were the same as the 1D spectra in figure 5 (B, C) of the main content of the manuscript.

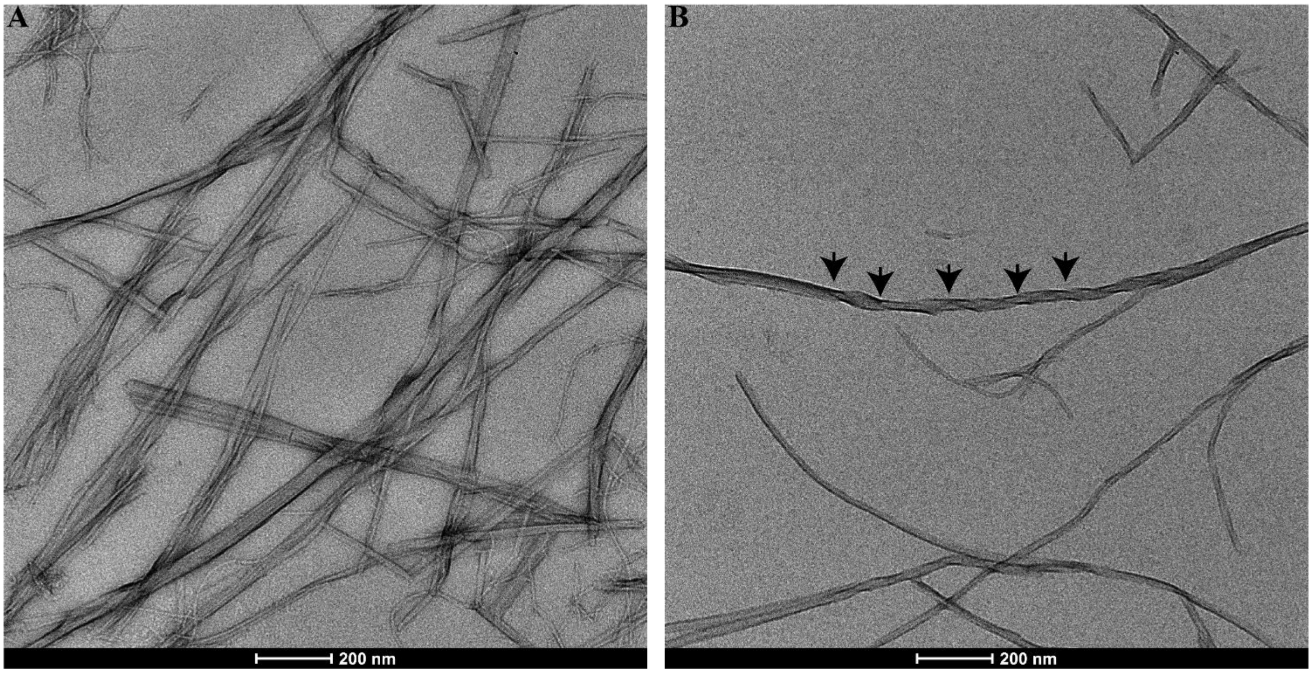

Supplementary Figure 3. TEM images of the 14P2 self-assembly formed at pH 6.0 with 33.4 mM calcium ions incubated for 2 days in different morphologies (A) and (B).

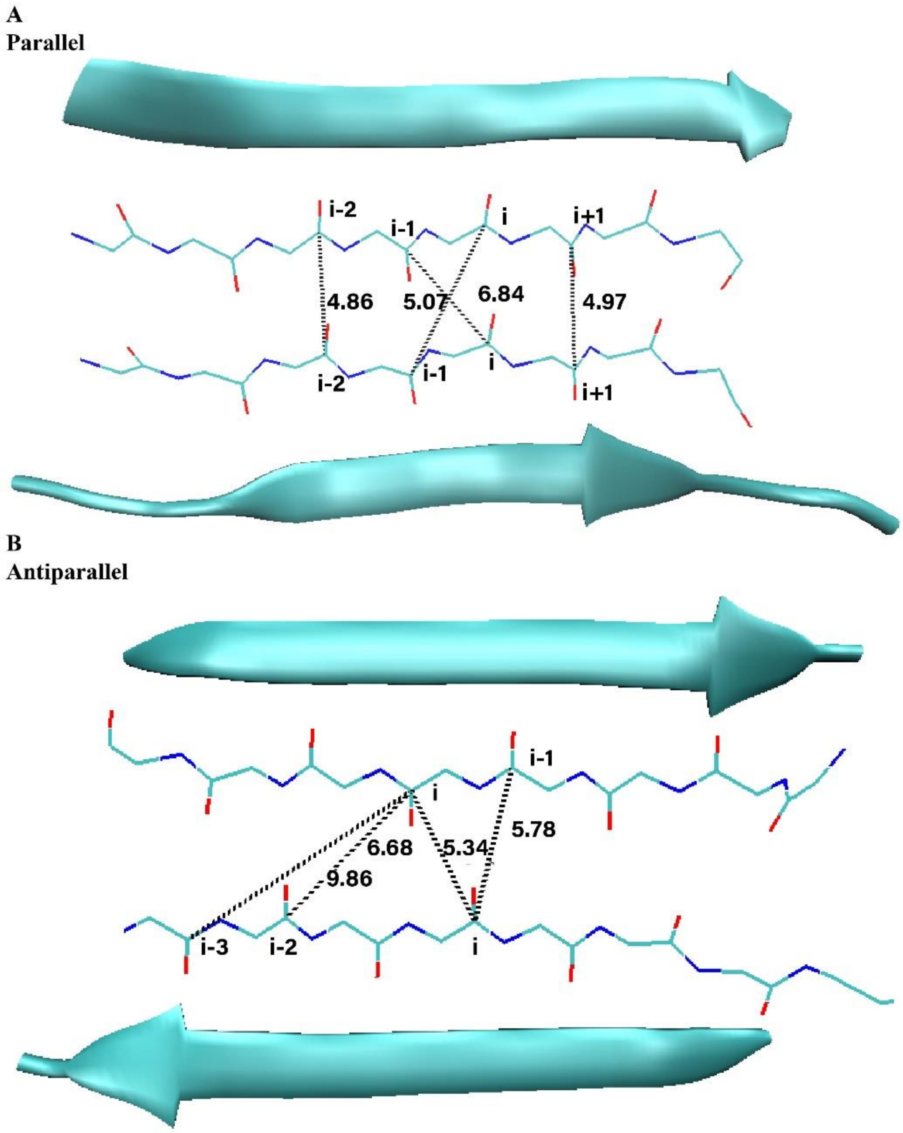

Supplementary figure 4. Aβ fibril structures showing specific pair of CO distances. (A) A parallel in-register Aβ fibril structure (PDB:2LMN). The two corresponding carbonyl carbon atoms at the adjacent peptides display a distance of 4.8-5.0 Å. The distances between residue i and i-1 are 5.07 Å or 6.84 Å. (B) An antiparallel Aβ fibril structure (PDB:2LNQ) with residue i aligned between the β-strands. The distance between residue i and i-1 is 5.78 Å. The distance between residue i and i is 5.34 Å. And the distance between residue i and i-2 is 6.68 Å.

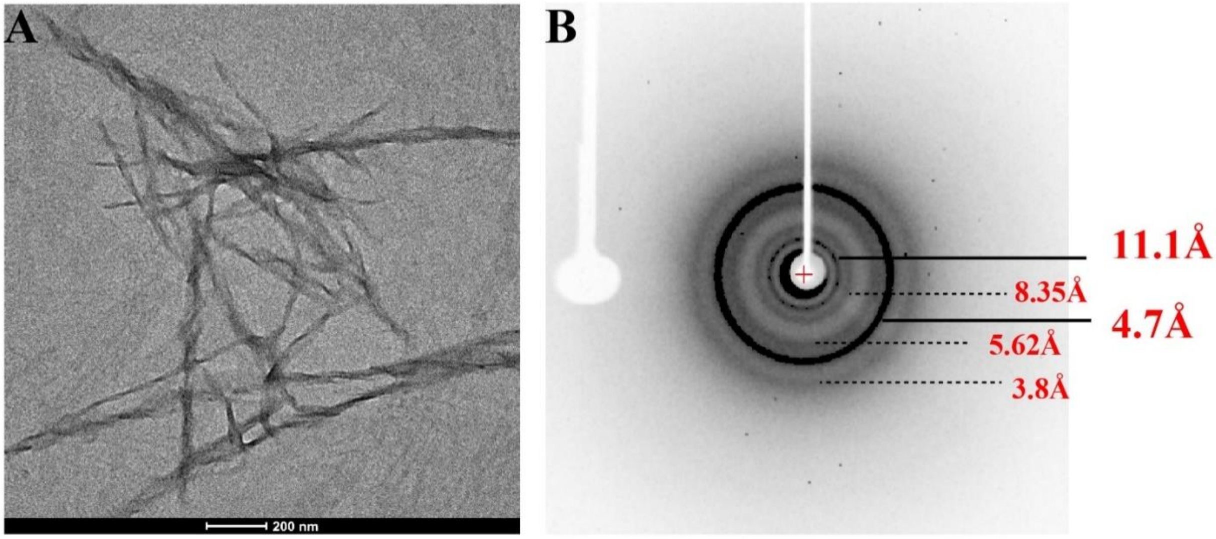

Supplementary figure 5. The TEM images and XRD of p14P2 assemblies formed at pH 7.5 in the presence of 3.3 mM calcium ions.

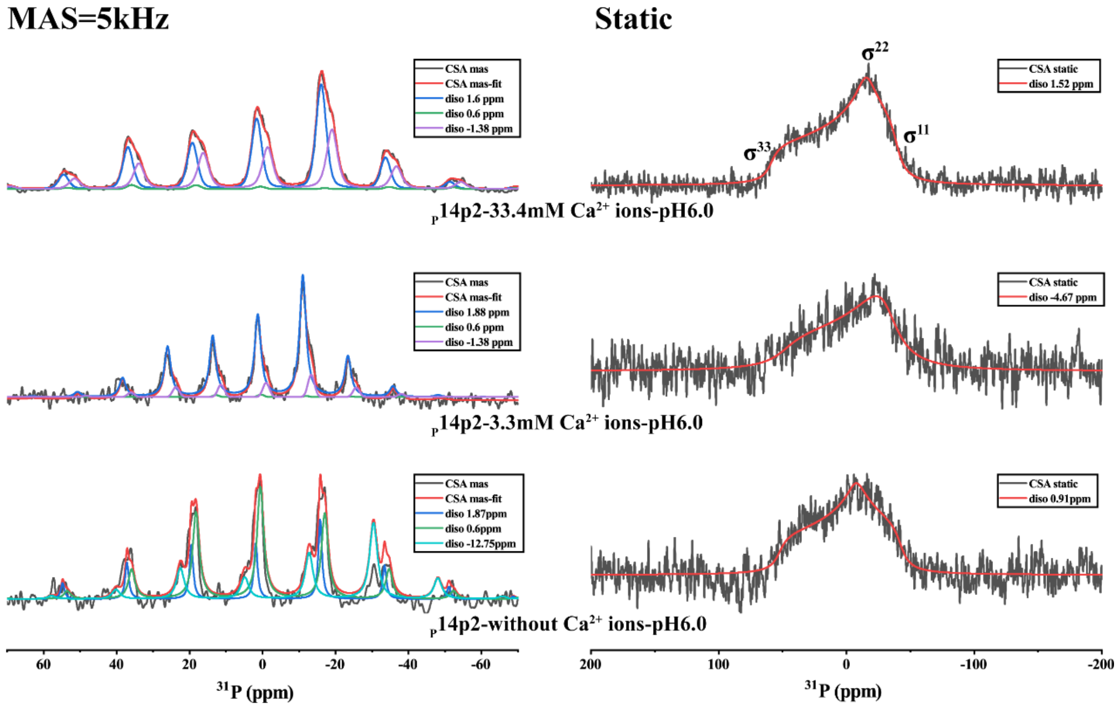

Supplementary Figure 6. ^{1}H-^{31}P CP-MAS and static NMR spectra of p14P2 self-assembly formed at different solution conditions. The CSA parameters were extracted from both MAS at 5 kHz spinning speed and the static condition, and the fitting parameters were shown in Supplementary Table 2.

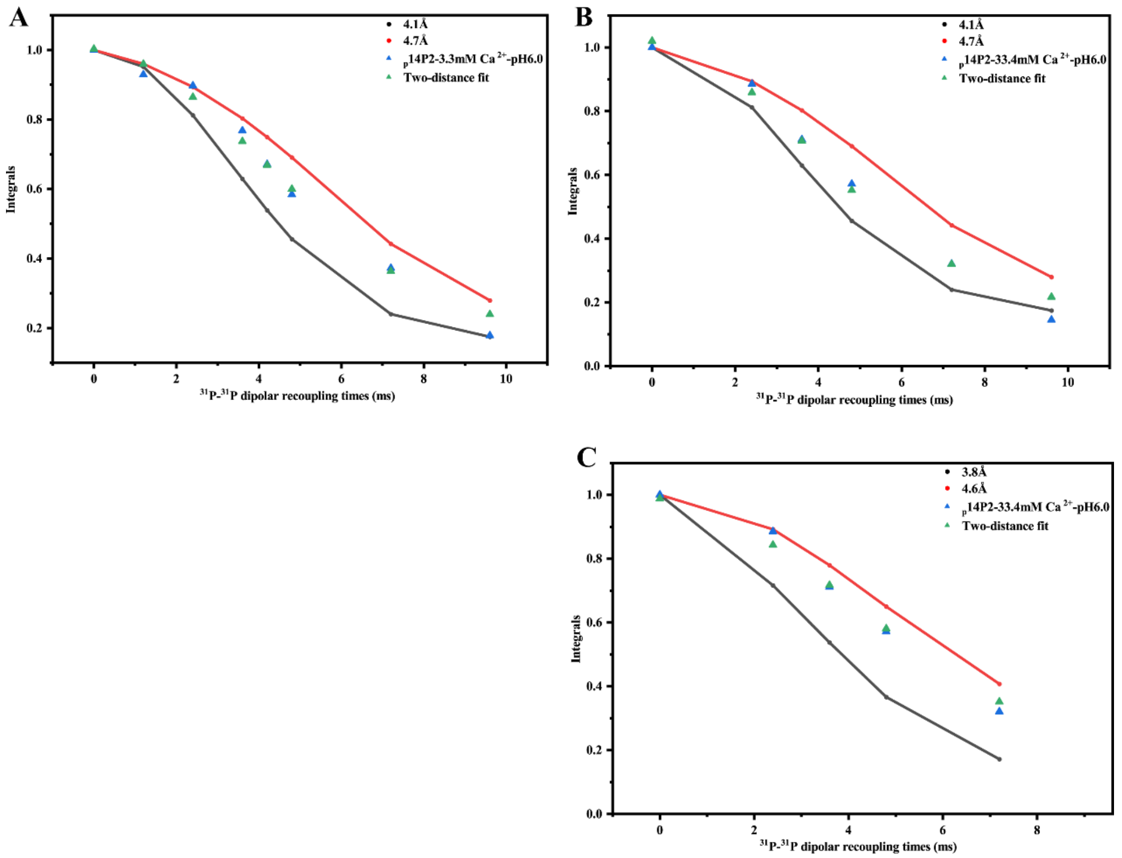

Supplementary Figure 7. Alternative fittings of ^{31}P PITHIRDS-CT experimental results, assuming two different conformations with two ^{31}P-^{31}P distances. (A) Two conformations with the ^{31}P-^{31}P distances 4.1 Å and 4.7 Å were used to fit the experimental results for p14P2 assemblies with 3.3 mM Ca^{2+}. The contribution of each conformation was 0.39 and 0.61, respectively. The deviation between the experimental and the fit values was expressed as RMSD, which was 0.0296; (B) Using the same two distances, the experimental decay was fit for p14P2 assemblies with 33.4 mM Ca^{2+}. The contribution of each conformation was 0.65 and 0.37, respectively and RMSD=0.0334; (C) Using two distances of 3.8 Å and 4.6 Å, the experimental decay was fit for p14P2 assemblies with 33.4 mM Ca^{2+}. The contribution of each conformation was 0.22 and 0.77, respectively and RMSD=0.0494.

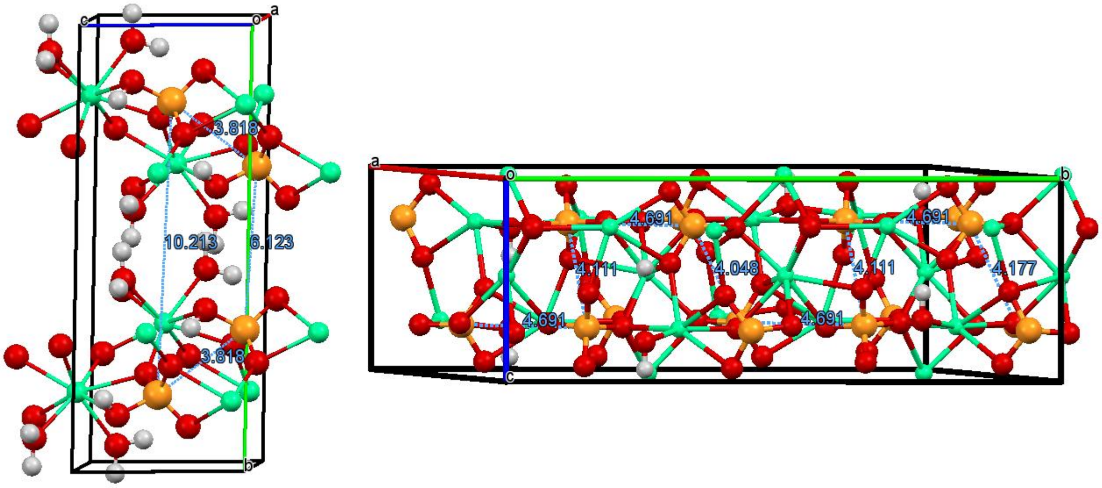

Brushite **HAP**

Supplementary Figure 8. The crystal structure of brushite and hydroxyapatite (HAP), showing ^{31}P-^{31}P distances. The color scheme: ^{31}P nuclei in orange, oxygen in red, calcium in green, hydrogen in white. The distance 3.818Å was shown for brushite along the c axis. Four distances about 4.1 Å were indicated for HAP along the c axis.

1.2 Supplementary Tables

Supplementary Table1. Solubility of calcium containing minerals.

Salt	pK_{sp}	Ion product (pI, high concentration)	Degree of saturation (DS, high concentration)	Ion product (pI, low concentration)	Degree of saturation (DS, low concentration)
Hydroxyl apatite $Ca_5(PO_4)_3(OH)$	58.5	45.84	12.66	51.13	7.37
Brushite $CaHPO_4$	6.6	5.01	1.58	6.61	-0.01

Degree of saturation= pK_{sp}-pI, pK_{sp}=-log10[Ksp], pI=-log_{10}[Ion product]. The bigger the DS value, the lower the solubility. High concentration refers the condition with total concentration of calcium ion =33 mM, total concentration of phosphates=21 mM. Low concentration refers the condition with total concentration of calcium ion =3.3 mM, total concentration of phosphates=2.1 mM. The calculation is based on a condition at 20 ℃ and pH=6 (Aoba and Moreno, 1987) .

Supplementary Table2. ${}^{31}P$ chemical shift anisotropy parameters extracted from ${}^{1}H$-${}^{31}P$ CP spectra in Figure 6 and Supplementary Figure 2 using DMFIT.

sample	dCS(ppm)	etaCS	σ^{11}(ppm)	σ^{22}(ppm)	σ^{33}(ppm)	span(ppm)	skew	σ^{iso}(ppm)	MAS
p14P2-33.4 mM Ca^{2+} -pH6	68.62	0.09	-35.67	-29.75	70.22	105.89	-0.89	1.6	15kHz
	-80.27	0.05	42.83	38.63	-79.67	122.5	0.93	0.6	
	63.5	0.85	-60.12	-6.14	62.12	122.25	-0.12	-1.38	
p14P2-3.3 mM Ca^{2+} -pH6	79.87	0	-38.52	-38.16	81.47	119.99	-0.99	1.6	
	-64.25	0.08	35.14	30.31	-63.65	98.79	0.9	0.6	
	79.93	0.3	-53.27	-29.42	78.55	131.81	-0.64	-1.38	
	88.51	0.11	-56.09	-46.74	81.35	137.44	-0.86	-7.16	
	67.86	0.93	-77.13	-14.23	56.11	133.23	-0.06	-11.75	
p14P2-without Ca^{2+} -pH6	42.28	0.55	-30.85	-7.69	44.15	75	-0.38	1.87	
	-35.86	0.01	18.74	18.33	-35.26	54	0.98	0.6	
	-31.92	0.2	13.65	7.37	-37.37	51.01	0.75	-5.45	
	58.01	0.34	-51.74	-31.77	45.26	96.99	-0.59	-12.75	
p14P2-33.4 mM Ca^{2+} -pH6	58.07	0.47	-41.17	-13.7	59.67	100.84	-0.46	1.6	5kHz
	-90.64	0.33	60.96	30.88	-90.04	151	0.6	0.6	
	58.8	0.69	-51.05	-10.51	57.42	108.48	-0.25	-1.38	
p14P2-3.3 mM Ca^{2+} -pH6	58.76	0.57	-44.13	-10.87	60.64	104.77	-0.37	1.88	
	-45.17	0.45	33.38	12.98	-44.57	77.95	0.48	0.6	
	63.06	0.66	-53.75	-12.07	61.68	115.42	-0.28	-1.38	
p14P2-without Ca^{2+}-pH6	59.93	0.77	-51.08	-5.11	61.8	112.88	-0.19	1.87	
	-44.27	0.99	44.6	0.87	-43.67	88.26	0.01	0.6	
	60	0.1	-45.75	-39.75	47.25	93	-0.87	-12.75	
p14P2-33.4 mM Ca^{2+} -pH6	57.45	0.42	-39.16	-15.25	58.97	98.13	-0.51	1.52	static
p14P2-3.3 mM Ca^{2+} -pH6	52.73	0.07	-32.9	-29.17	48.06	80.96	-0.91	-4.67	
p14P2-without Ca^{2+} -pH6	51.64	0.67	-42.24	-7.59	52.54	94.78	-0.27	0.91	

dCS and etaCS are asymmetry and anisotropy parameters of the CSA tensor, respectively (dCS =(δ_{33}-δ_{iso}), etaCS=(δ_{22}-δ_{11})/(δ_{33}-δ_{iso})). The span describes the maximum width of spectrum (span= δ_{11}-δ_{33}). The skew is a measure of the amount and orientation of the asymmetry of the tensor,

which depends on the position of δ_{22} with respect to δ_{iso} (skew=3(δ_{22}-δ_{iso})/span)(Addison et al., 2013; Herzfeld and Berger, 1980). When the phosphoserine interacts with the monovalent (Na^+) or divalent ions (Ca^{2+}) at pH6.0, a negative sign of the skew value will be shown, indicating the coordination interactions of phosphoserine with the ions. The organic phosphate group on the p14P2 peptide assemblies has multiple conformations, indicated by multiple peaks. The peaks centered at 1.6 ppm and -1.38 ppm show the negative skew values, supporting the calcium binding.

References

Aoba, T., and Moreno, E.C. (1987). The enamel fluid in the early secretory stage of porcine amelogenesis: Chemical composition and saturation with respect to enamel mineral. *Calcified Tissue International* 41(2)**,** 86-94. doi: 10.1007/BF02555250.

Addison, J.B., Ashton, N.N., Weber, W.S., Stewart, R.J., Holland, G.P., and Yarger, J.L. (2013). β-Sheet nanocrystalline domains formed from phosphorylated serine-rich motifs in caddisfly larval silk: a solid state NMR and XRD study. *Biomacromolecules* 14(4)**,** 1140-1148. doi: 10.1021/bm400019d.

Herzfeld, J., and Berger, A.E. (1980). Sideband intensities in NMR spectra of samples spinning at the magic angle. *The Journal of Chemical Physics* 73(12)**,** 10. doi:10.1063/1.44013
